# Role of Gut Microbiota and Probiotics in Colorectal Cancer: Onset and Progression

**DOI:** 10.3390/microorganisms9051021

**Published:** 2021-05-10

**Authors:** Edgar Torres-Maravilla, Anne-Sophie Boucard, Amir Hossein Mohseni, Sedigheh Taghinezhad-S, Naima G. Cortes-Perez, Luis G. Bermúdez-Humarán

**Affiliations:** 1 Micalis Institute, Université Paris-Saclay, INRAE, AgroParisTech, 78350 Jouy-en-Josas, France; edgar.torres-maravilla@inrae.fr (E.T.-M.); Anne-Sophie.Boucard@inrae.fr (A.-S.B.); 2 Department of Microbiology, Faculty of Basic Sciences, Science and Research Branch, Islamic Azad University, Tehran 1477893855, Iran; amho.mohseni@gmail.com (A.H.M.); taghinezhad.m@gmail.com (S.T.-S.); 3 Université Paris-Saclay, INRAE, AgroParisTech, UMR 0496, 78350 Jouy-en-Josas, France; naima.cortes-perez@inrae.fr

**Keywords:** microbiota, probiotics, lactic acid bacteria, cancer, colorectal cancer

## Abstract

The gut microbiota plays an important role in maintaining homeostasis in the human body, and the disruption of these communities can lead to compromised host health and the onset of disease. Current research on probiotics is quite promising and, in particular, these microorganisms have demonstrated their potential for use as adjuvants for the treatment of colorectal cancer. This review addresses the possible applications of probiotics, postbiotics, synbiotics, and next-generation probiotics in colorectal cancer research.

## 1. Introduction

Currently, colorectal cancer (CRC) is the third most common cancer worldwide, with more than one million new cases and 600,000 deaths each year [1]. There are two types of CRC: colitis-associated, caused by the presence of a mutation in the *TP53* gene, and sporadic, caused by a mutation in the adenomatous polyposis coli (*APC*) gene. However, genetic factors play a relatively minor role in cancer development (<10% to 30%); instead, cancer risk is greatly influenced by extrinsic (e.g., environmental) factors such as infectious agents, antibiotic administration, high-fat diets, red meat consumption, and a deficiency in fiber intake [2,3]. All of these components are known to alter gut microbiota and induce dysbiosis [4], defined as perturbations in commensal communities that can lead to the deficient education of the host immune system and the subsequent development of immune-mediated diseases. Dysbiosis can be categorized into three types: (i) loss of beneficial species, (ii) expansion of pathobionts or potentially harmful species, and (iii) loss of overall microbial diversity [5]. All three types of dysbiosis have been observed in CRC patients.

One of the means by which healthy gut microbiota may exert their anticancer effects is through the beneficial metabolites they produce, which can have antioxidant and anti-inflammatory properties, regulate bowel barrier function, act as vitamins, and represent a source of energy. Instead, the gut microbiota of CRC patients can have direct pro-tumorigenic effects; for example, a gavage of fecal samples from CRC patients was observed to promote intestinal carcinogenesis in both germ-free and conventional mice [6]. Recent research into probiotics, and into microbiota more generally, has yielded promising outcomes and has demonstrated the serious potential of these assemblages as co-adjuvants in colon cancer therapies. This review addresses the current state of research on the role of the gut microbiota, as well as the efficacy of probiotics, postbiotics, synbiotics, and next-generation probiotics (NGPs), in CRC treatment and prevention.

## 2. Gut Microbiota

The microbiota, also referred to as the microflora, is defined as the entire population of microbes present within the human body, which principally includes bacteria, archaea, and eukarya [7], as well as viruses [8]. The quantity of these microorganisms is staggering. The human gastrointestinal tract alone can host nearly 100 trillion (10^14^) microorganisms [9], a number nearly three times greater than the total number of cells in the entire human body (recently recalculated as 3.7 × 10^13^) [10]. From a physiological point of view, the microbiota makes up about 2% of an adult’s body mass, almost equivalent to the size of the human brain or liver [11], which has led some researchers to refer to the microbiota as the forgotten human organ [12,13]. Many essential body processes require the presence of these diverse and numerous microorganisms, as they provide the host with nutrients, metabolize indigestible compounds, and can help in the defense against colonization by opportunistic pathogens, as well as possess immune-modulatory properties [14].

With the use of next-generation DNA sequencing technologies and metagenomic analysis, it has been shown that the gut microbiota of vertebrates is composed of approximately 500–1000 different bacterial species, of which 98% are represented by two dominant phyla, Bacteroidetes and Firmicutes [15,16,17]. One of the most surprising discoveries has been the fact that the number of genes in the gut microbiota is approximately 100 times larger than the human genome and appears to represent a co-evolutionary relationship [17]. Due to the numerous interactions among different microbial species, human host cells, and the external environment, the microbiota can also be conceptualized as being a dynamic ecological community [18]. In this sense, a dynamic equilibrium of the microbiota in the human body is necessary for health, which can be disrupted by environmental factors and external stimuli such as the use of antibiotics, illness, stress, aging, bad dietary habits, and lifestyle [19]. These alterations frequently result in microbial imbalances—dysbiosis—with direct links to multiple pathological conditions [20].

For example, between 2018 and 2021, a search for “microbiota dysbiosis” and “diseases” in PubMed returned 5,617 published articles describing links between dysbiosis of the gut microbiota and diseases such as obesity [21], autism spectrum disorders [22], cardiovascular diseases [23], diarrhea [24], alcoholic liver disease [25], acute-on-chronic liver failure [26], arthritis [27], lung diseases [28], autoimmune diseases [29], lupus erythematosus [30], coeliac disease [31], intestinal inflammatory diseases such as Crohn’s disease, colitis and irritable bowel syndrome (IBD) [32], and colorectal cancer [33] (these account for 531 citations by themselves).

## 3. CRC and Gut Microbiota

**Figure 1:**
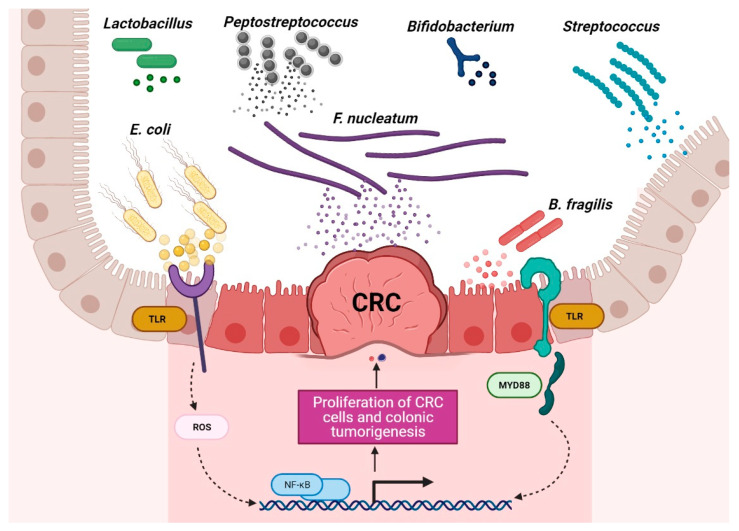
Schematic of the roles of probiotic and harmful bacteria in CRC context. Disruption of the gut microbiota balance is associated with CRC development, and regulation of probiotic bacteria is associated with CRC remission. (The figure was created with Biorender.com).

Compared to healthy individuals, CRC patients harbor a distinct mucosa-associated microbiota. For instance, the effect of CRC on the microbiota is generally characterized by an increase in microbial diversity that seems to progress with cancer development—late CRC samples (stage III and stage IV) generally display higher richness levels than early CRC samples do (stage I and stage II) [34]. At the phylum level, increasing numbers of Bacteroidetes, Firmicutes, and Fusobacteria and decreasing numbers of Proteobacteria in mucosa-associated microbiota are observed. At the genus level, CRC progression tends to be associated with the proliferation of *Fusobacterium, Peptostreptococcus, Streptococcus*, and *Ruminococcus* and a decline in *Lactobacillus* and *Granulicatella* [34,35] (Figure 1). Differences in mucosa-associated microbiota can also be observed before the appearance of a cancerous state. For example, Flemer et al. [36] detected significant differences between the mucosa-associated microbiota from subjects with polyps and from healthy controls, suggesting that the gut microbiota is involved in cancer development from a very early stage. Another large-cohort multi-omics dataset indicated that shifts in the microbiome and metabolome occur from the very early stages of the development of colorectal cancer, which could be of possible etiological and diagnostic importance [37]. Accordingly, it has been reported that a long-term exposure (≥2 months) to antibiotics in early to middle adulthood is associated with an increased risk for colorectal adenoma at the age of 60 [38]. Interestingly, the microbiota alterations observed in CRC patients are not restricted to the tumor site; they can also be seen in the surrounding healthy tissue. Indeed, in multiple cohorts, the mucosa-associated microbiota from paired samples of tumor tissue and nearby nontumor mucosa was similar with regard to both individual taxa and the overall microbiota composition [34,35,39,40].

**Figure 2:**
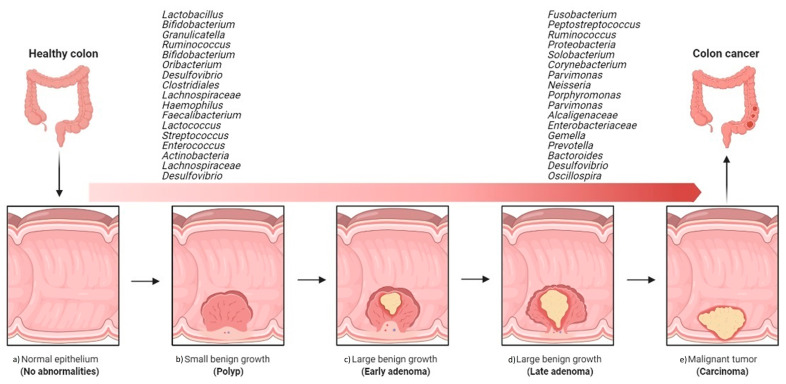
Overview of the implications of gut bacteria in the development and progression of colorectal cancer. Shift from healthy intestine to CRC intestine: (**a**) normal epithelium, (**b**) poly P (small benign growth), (**c**) early adenoma, (**d**) late adenoma, and (**e**) carcinoma. (The figure was created with Biorender.com).

A comparative study of coupled fecal and mucosal samples demonstrated that, although fecal microbiota only partially reflects the community at the mucus layer, differences due to CRC are still evident in fecal samples [36]. Thus, this noninvasive approach is the most commonly used sampling method in gut microbiome studies. Fecal samples from CRC patients differ significantly from those of healthy subjects, both in microbial richness and community composition. In fecal samples, CRC development is usually associated with an increase in pro-inflammatory or pathogenic species belonging to phyla Proteobacteria and Fusobacteria and a decrease in beneficial species of phylum Firmicutes [41]. As observed with the mucosa-associated microbiota, the fecal microbiota of CRC patients is dynamic, with characteristic changes during cancer progression. In a Chinese cohort, fecal samples from healthy individuals were dominated by Bacteroidetes and Firmicutes, the abundance of which decreased with progression along the polyp–adenoma–carcinoma sequence. In contrast, the abundance of Proteobacteria was noted to increase with colon cancer development [42]. Such shifts have even been seen in the relative abundance of individual bacterial taxa. In particular, Firmicutes, Actinobacteria, Lachnospiraceae, and the genus *Desulfovibrio* have been shown to be specific to early-stage CRC, while the genera *Solobacterium, Peptostreptococcus, Corynebacterium, Parvimonas, Neisseria, Porphyromonas, Gemella*, and the families Alcaligenaceae and Enterobacteriaceae appear to be associated with malignancy [37,43,44,45] (Figure 2).

### 3.1. Inflammation and CRC

**Figure 3:**
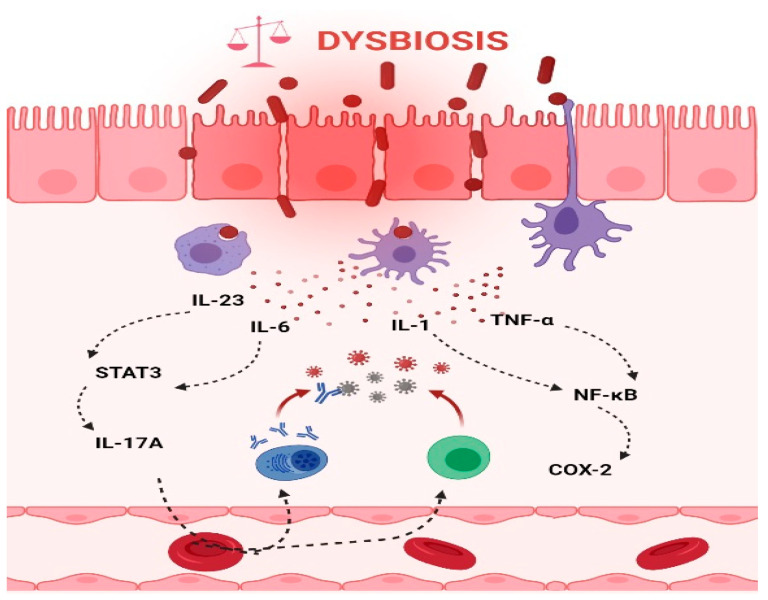
Intestinal inflammation caused by gut microbiota contributes to the onset of CRC. Dysbiotic bacteria can elicit immune imbalances and facilitate the translocation of gut microbiota and/or its metabolites due to a leaky gut to the tissues and systemic circulation. These events may lead to the stimulation of an inflammatory state and ultimately to the development of CRC. Thus, the production of IL-6 and IL-23, in turn, trigger the expression of IL-17A and contribute to the development of CRC through STAT3 activation. In addition, TNF-α and IL-1 promote pro-inflammatory and pro-tumorigenic activities of COX-2 that stimulate growth and angiogenesis and inhibit apoptosis in CRC. (The figure was created with Biorender.com).

Chronic inflammation is an established risk factor for CRC, as patients with inflammatory bowel diseases (IBD) consistently have a higher risk than the general population of developing CRC [46,47]. Correspondingly, an increase in pro-inflammatory species has been repeatedly reported in CRC patients (Figure 3). The most prevalent and most described bacterium in CRC fecal and mucosa-associated microbiota is *Fusobacterium nucleatum* [45], which, in murine models, increases the proliferation of CRC cells and colonic tumorigenesis by activating TLR4 signaling to NF-κB, thus promoting the infiltration of specific pro-inflammatory myeloid cell subsets into tumors [48,49,50]. Interestingly, a recent study found that more than 40% of CRC patients exhibited identical strains of *F. nucleatum* in both tumor and saliva samples, suggesting that *F. nucleatum* in CRC originates from the oral cavity [51]. In this, *F. nucleatum* does not appear to be alone; meta-analyses of geographically and technically diverse cohorts have identified several oral commensal and pathogenic bacteria that are significantly enriched in CRC samples, including members of the genera *Fusobacterium, Porphyromonas, Parvimonas, Peptostreptococcus, Gemella, Prevotella*, and *Solobacterium*. Taken together, these results reinforce the hypothesis of an oral–gut translocation route that is associated with inflammation and CRC [39,52,53,54,55]. Other examples of well-known pro-inflammatory species with links to CRC are colibactin-producing *Escherichia coli*, which enhances inflammation and the production of reactive oxygen species (ROS) in tumors in early-stage CRC [56], and enterotoxigenic *Bactoroides fragilis*, which mediates inflammation through the Th17 response and NF-κB activation, thus inducing myeloid cell-dependent distal colon tumorigenesis [57]. The increase in pro-inflammatory species observed in CRC correlates with a reduction in anti-inflammatory species belonging to the beneficial genera *Ruminococcus, Bifidobacterium, Lachnospira, Oribacterium, Desulfovibrio, Clostridiales*, and *Lactobacillus* [44,55,58]. Furthermore, the alterations observed in CRC with respect to microbiota composition may also translate into changes in metabolite concentrations. Specifically, a metabolomic study detected a direct association between significantly lower abundances of Clostridia and Lachnospiraceae in CRC and reduced quantities of the metabolites p-aminobenzoate and conjugated linoleic acid, which are known to exhibit anti-inflammatory and anti-cancerogenic properties [59].

### 3.2. DNA Damage and CRC

Certain members of the CRC-enriched microbiota are able to directly induce DNA damage to colonic epithelial cells. For example, some strains in the CRC-associated family Enterobacteriaceae produce ROS and colibactin, a toxin responsible for oncogenic mutations in host colonic epithelial cells [60,61]. The pivotal role of this toxin in carcinogenesis was confirmed by the finding that colibactin-producing *E. coli* promote tumorigenesis in Apc^Min/+^; IL10^−/−^ mice in a colibactin-dependent manner [62]. A recent study identified a distinct mutational signature of genotoxic *pks*+ *E. coli* in human intestinal organoids, and this same signature was also detected in human CRC genomes. This suggests that the underlying mutational process may be the direct result of past exposure to bacteria that carried the colibactin-producing *pks* pathogenicity island [60]. However, *E. coli* is far from the only species with this capability. Molecular studies of *F. nucleatum* have discovered the virulence protein FadA and its involvement in the transformation of epithelial cells and the promotion of colon tumorigenesis [63]. A meta-analysis of CRC fecal metagenomes confirmed the significant enrichment in both the colibactin-producing gene cluster *pks* and the *F. nucleatum* adhesin *fadA* [53]. In vitro, enterotoxins produced by *B. fragilis* have been associated with DNA damage and genomic instability [64], while, in mice, *Peptostreptococcus anaerobius*, another CRC-enriched species, was shown to interact with TLR2/4 receptors on host cells to induce ROS production, increasing cholesterol biosynthesis and activating pro-oncogenic factors and CRC-promoting pathways [65]. CRC patients present increased amounts of sulphate-reducing bacteria belonging to genus *Desulfovibrio* [37,44], which explains the elevated levels in late-stage CRC of dissimilatory sulfate reductase subunit A, a gene responsible for the production of genotoxic hydrogen sulfide [37]. Multiple metabolomic analyses have reported increases in polyamines, such as putrescine and cadaverine, in CRC fecal samples compared to healthy controls [41,64]; in particular, the polyamine spermidine is known to promote colibactin-associated genotoxicity [66]. Finally, evidence from whole metagenomics analysis has linked the relative abundance of some members of the CRC microbiota with the methylation or demethylation of host genes, indicating that epigenome dysregulation may be another means by which CRC-associated dysbiosis promotes colon carcinogenesis [58]. For example, the *B. fragilis* toxin is able to induce epigenetic changes in vitro in HT-29 colon epithelial cells. The toxin alters the expression of specific genes, the accessibility of certain transcription factor binding sites, and the coordination between regions with different degrees of methylation, which, together, increases the risk of colon tumorigenesis [67].

### 3.3. Short-Chain Fatty Acids and CRC

Short-chain fatty acids (SCFAs) are the primary end-products of the fermentation of polysaccharides and nondigestible carbohydrates that remain available to the gut microbiota. Butyrate, acetate, and propionate are the most abundant SCFAs. Butyrate, in particular, has a remarkable array of colonic health-promoting and antineoplastic properties; along with being the preferred energy source for colonocytes, it maintains mucosal integrity, reduces pro-inflammatory cytokines, and induces apoptosis in CRC cell lines [68]. Compared to healthy controls, the fecal microbiota of patients with CRC and advanced colorectal adenoma demonstrates significant reductions in the abundance of butyrate-producing bacteria [55,69], and these reductions are dependent on CRC progression. Notably, the abundance of *Oscillospira* declines in the transition from advanced adenoma to stage 0 CRC, whereas levels of *Haemophilus* decrease in the transition from stage 0 to early-stage CRC [45]. A meta-analysis of fecal metagenomes confirmed a significant decrease in the carbohydrate-degradation genes responsible for SCFA production in CRC [53]. These changes in the microbiome and metagenome coincide with a decrease in butyrate concentration in CRC patients [41,69].

### 3.4. Bile Acid Metabolism and CRC

Primary bile acids are synthesized in the liver, conjugated to taurine or glycine, and released in the gut. Upon reaching the colon, bile acids are deconjugated by bile salt hydrolases of the gut microbiota and are subsequently transformed into dangerous secondary bile acids by 7α-dehydroxylating bacteria [70]. Alterations in this process have been associated with CRC. Metabolomic profiling confirmed the presence of elevated levels of secondary bile acids, including deoxycholic acid (DCA), in adenomas and/or intramucosal carcinomas [37,71]. In mice, DCA has been found to induce alterations in the gut microbiota that are accompanied by impairments in the intestinal barrier, low-grade inflammation, and colonic tumors [72,73]. DCA-induced dysbiosis is characterized by an increased abundance of pathogens and a decreased abundance of probiotics, and this shift in microbial community structure can be sufficient by itself to cause disease independent of DCA treatment [73]. Instead, the secondary ursodeoxycholic acid (UDCA), known for its anti-carcinogenic properties, is less abundant in CRC patients [69,74].

Generally speaking, a high-fat and low-fiber diet has long been known to represent a risk factor for CRC; specifically, this diet correlates with lower levels of colonic SCFAs and higher levels of colonic secondary bile acids and mucosal proliferative biomarkers of cancer risk [75]. Interestingly, directed dietary changes (switch from high-fat/low-fiber to low-fat/high-fiber diet and vice versa) resulted in remarkable reciprocal changes in mucosal biomarkers of cancer risk, with an increased saccharolytic fermentation and butyrogenesis and a suppressed secondary bile acid synthesis in the low-fat/high-fiber diet group [75].

## 4. Microbiota Biomarkers for CRC Diagnosis

Unlike the well-described causal role of *Helicobacter pylori* in gastric ulceration and cancer, a specific and universal microorganism that triggers CRC has not been identified. Instead, the evidence points to a shift in microbial composition, accompanied by changes in microbial gene abundance and microbe-associated metabolites, which all tend to be proportionate with the degree of malignancy. It is not clear whether these species and metabolites directly cause tumorigenesis; if not, the culprit may be the microenvironment created by these structural shifts, which then promotes inflammation, proliferation, and cancer progression [37,54]. Regardless, the identification of reproducible microbial biomarkers for CRC may enable the design of noninvasive tools for CRC diagnosis. For example, CRC patients demonstrate enrichments in pro-inflammatory *F. nucleatum* and reduced levels of beneficial *Faecalibacterium prausnitzii* and *Bifidobacterium*. In vitro, *F. nucleatum* displays strong bacteriostatic activities against these probiotic bacteria. Thus, the ratio of *F. nucleatum*/*F. prausnitzii* and *F. nucleatum*/*Bifidobacterium* has been proposed as a biomarker for screening early CRC [76]. Going further, a multi-cohort analysis of gut metagenomes identified seven CRC-enriched bacteria—*B. fragilis, F. nucleatum, Porphyromonas asaccharolytica, Parvimonas micra, Prevotella intermedia, Alistipes finegoldii*, and *Thermanaerovibrio acidaminovorans*—which performed well in distinguishing CRC samples from controls across different populations and may, thus, have potential for universal use for noninvasive CRC diagnosis [52]. The association between CRC and *Streptococcus gallolyticus* subsp. *gallolyticus* (formerly known as *S. bovis* type I) has also been reported [77,78,79]. A meta-analysis performed by Boleij et al. [78] reported an association between the infection from *S. gallolyticus* subsp. *gallolyticus* and CRC in 65% of cases. The strong association between *S. bovis* infection and colonic adenomas and carcinomas has led to the speculation about the possible oncogenic (driver or passenger) role of this bacterium. Indeed, the oral administration of *S. gallolyticus* in a mouse model of azoxymethane (AOM)-induced CRC led to a higher number of tumors, higher level of dysplasia, and increased cell proliferation and β-catenin levels in colon crypts as compared to control mice treated with *L. lactis* strain [79]. In any case, every infection by any of these subspecies should lead to a colonoscopy diagnosis and should not be underestimated, and a complete intestinal examination is highly recommended for patients presenting a *S. bovis* bacteremia, especially when S. *gallolyticus* subsp. *gallolyticus* is involved (biotype I) [80].

A metabolomic analysis of a Chinese cohort identified 20 gene markers that significantly differentiated CRC-associated and control samples. Four of these markers—butyryl-CoA dehydrogenase from *F. nucleatum*, two transposases from *P. anaerobius*, and RNA polymerase subunit β (*rpoB*) from *P. micra*—were found to also be present in Danish, French, and Austrian cohorts. This suggests that even though human populations may differ with respect to the structure of gut microbial communities, there may be certain universal signatures of CRC-associated microbial dysbiosis [54]. Furthermore, levels of gut microbiota-derived metabolites, such as SCFAs, bile acids, and protein-derived metabolites, have been repeatedly associated with CRC progression and have been proposed as complementary biomarkers for the early screening of CRC [81]. In addition to providing diagnostic markers, analysis of the gut microbiota might also help in CRC prognosis. For example, *F. nucleatum* enrichment in cancer tissue is associated with a shorter survival and may, therefore, act as a potential prognostic marker [82]. Another potential method for the noninvasive detection/diagnosis of CRC could rely on the correlation of metabolomic signatures from fecal samples. Recently, analyses of stools have led to the identification of several relevant fecal metabolites. In addition to SCFAs (acetate and butyrate), metabolites such as xenobiotics, heme, peptides/amino acids (proline and cysteine), vitamins, and co-factors all demonstrated alterations in CRC samples [37,83]. In addition, CRC metagenome analyses have highlighted enrichments in protein and mucin catabolism genes, and depletions in carbohydrate degradation genes and bile acid genes, which could be used as signatures for CRC diagnostics. For example, intestinal *Clostridium* species are known to contribute to the conversion of primary to secondary bile acids via the 7α-dehydroxylation pathway encoded in the *bai* operon. *Bai* was found to be highly enriched in stools from CRC patients and may represent a possible CRC biomarker. However, *bai* enrichment is also a consequence of a diet rich in meat and fat; therefore, more studies are necessary to elucidate any potential biomarker function [53]. In general, further research is needed to identify microbes that are universally associated with CRC for use in early noninvasive diagnosis and prognosis.

## 5. Other Microbiota Microorganisms

While the role of the bacterial microbiota in CRC has been extensively studied, little data are available regarding other members of the intestinal community, such as viruses, fungi, and archaea [84]. With respect to fungi, Richard et al. [40] noted no differences in mucosal samples from CRC patients compared to healthy controls. In fecal samples, however, fungal dysbiosis has been observed, with an increased ratio of Basidiomycota to Ascomycota in CRC patients. Ecological analysis revealed a higher number of co-occurring correlations among fungi and more co-exclusive correlations between fungi and bacteria in CRC compared with control samples, indicating possible roles in colorectal carcinogenesis for synergistic intra-fungal relationships and antagonistic bacterial–fungal associations [85]. However, an indirect protective role of fungi was also recently reported in mice, as fungal commensals were found to induce IL-18 and thus inhibit colitis-associated CRC [86].

Regarding enteric archaea, alterations in community composition have been observed during tumorigenesis. In particular, fecal samples from patients with CRC demonstrated significant enrichments in halophilic archaea and depletions in methanogenic archaea. Furthermore, CRC-associated halophiles were positively associated with the oncogenic bacterium *B. fragilis* and were negatively correlated with butyrate-producing *Clostridium* species [87].

Little is known about the viral component of the CRC-associated microbiome. Two recent studies reported an increase in the diversity of the gut bacteriophage community in patients with CRC. The authors described altered interactions between bacteriophages and oral bacterial commensals, suggesting that the bacteriophages modulate the bacterial community and, through those interactions, indirectly influence the bacteria that drive colorectal cancer progression [88,89]. A more thorough understanding of the virome, of which bacteriophages are an important part, is crucial to understand the etiology and progression of CRC. Indeed, viruses may play a pivotal role from a very early stage; broadly infectious phages in the colon can lyse, and thereby disrupt the biofilms of, the existing bacterial communities in the intestinal mucosa. This alteration then enables the growth of oncogenic bacteria that are able to transform epithelial cells and disrupt tight junctions to infiltrate the epithelium, thereby provoking an inflammatory immune response [89].

Although extensive research remains to be performed, these findings suggest that dysbiosis in fecal communities of archaea, fungi, and viruses may contribute, together with or in addition to bacteria, to colon tumorigenesis.

## 6. Probiotics in CRC

**Figure 4:**
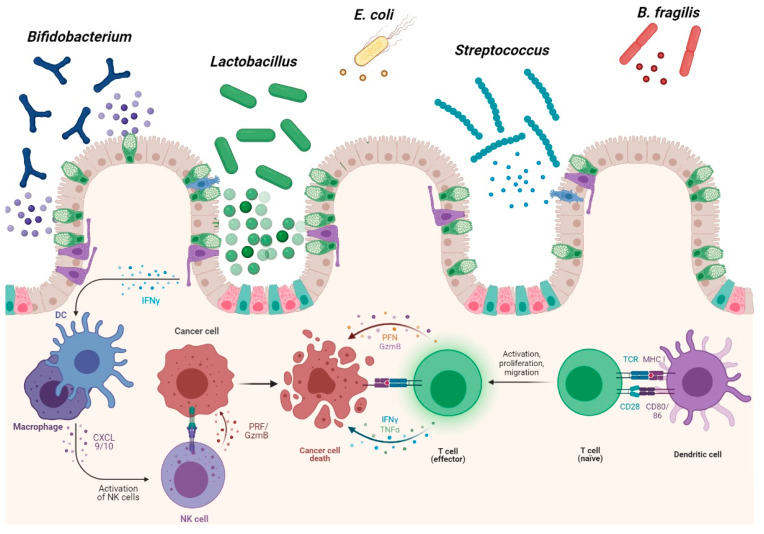
Positive effects of microbiota and probiotics in CRC. Some probiotic strains and their metabolites are able to enhance the intestinal immune system by inhibiting the development of cancer cells. Probiotics promote macrophage and dendritic cell differentiation, enhancing the activation of cytotoxic T-cells and NK cells. (The figure was created with Biorender.com).

Currently, a broad search is ongoing for alternatives to help in the treatment of cancer. As mentioned above, dysbiosis of the microbiota is closely associated with cancer risk and CRC development. This dysbiosis can be redressed by probiotic strains, which, in effect, shift the composition of the microbiota toward more favorable species. Probiotics are defined as “live microorganisms which, when administered in adequate amounts, confer a health benefit on the host” [90]. Commercial and medicinal probiotics have demonstrated potentials beyond simply modulating the gut microbiota; they have become attractive and promising agents of host–microbiome modulation therapies for several diseases, including CRC (Figure 4).

Several studies have documented both their safety and effectiveness in enhancing the action of chemotherapy in the fight against cancer, as well as attenuating the side-effects of conventional treatments; there is some evidence to support the hypothesis that probiotics may also be able to minimize the development and progression of CRC by mitigating the aggressiveness of tumors. At present, probiotics have been extensively adopted by adherents of ‘wellness’ lifestyles, who consume foods that, in addition to their nutritional value, offer benefits to improve the overall well-being. To meet this demand, many commercial products containing probiotic microorganisms have been developed and made available throughout the world. Such products typically make use of the genera *Lactobacillus, Bifidobacterium, Lactococcus, Streptococcus*, and *Enterococcus*. However, other strains, such as *Bacillus, Saccharomyces*, and next-generation probiotics (NGPs), are currently under study for use in treating CRC (Table 1).

The beneficial effects of probiotics have been demonstrated both in vitro and in preclinical trials, particularly for species of *Lactobacillus*, which has been, by far, one of the most documented genera. Many studies have examined the ways in which probiotic strains affect or interact with pathogenic microorganisms that contribute to the development of CRC, such as *Helicobacter pylori, Salmonella, B. fragilis, F. nucleatem*, and some strains of *E. coli*. These pathogens are capable of degrading the gut and releasing highly toxic compounds, thus compromising the balance of intestinal homeostasis. Probiotics fight against the proliferation of harmful microorganisms through lowering the pH of the environment, producing bacteriocins, and reducing the level of pro-carcinogenic enzymes [120]. There has been a particular focus on infection by *H. pylori*, which can potentially increase the risk of colorectal cancer. Specifically, many studies have reported a link between *H. pylori* infection and increased serum levels of gastrin; hypergastrinemia is associated with rectal cell proliferation and stimulates the growth of colorectal cancer cells and the development of colon adenomas [121]. In human gastric epithelial cells, *Lactobacillus acidophilus* and *Lactobacillus bulgaricus* were found to protect against the negative effects of *H. pylori* in two ways. First, both strains inhibited *H. pylori* adherence through the production of acetic acid and other bactericidal substances. Then, *L. bulgaricus* was found to prevent TLR4/NF-κB signaling and, thus, the production of the IL-8 pro-inflammatory cytokine that can lead to chronic inflammation as a result of *H. pylori* infection [94]. Based on the results of meta-analyses, though, it does not seem that probiotic therapies can be considered alternatives to anti-*H. pylori* treatment. Rather, the association of probiotics with standard antibiotic treatment could significantly improve the eradication rates of *H. pylori*, as well as decrease the side-effects of current medication therapy [122].

Recent studies have provided strong evidence to support a causative role for colibactin, a genotoxin of unknown structure, in CRC [123]. Colibactin is produced by *E. coli*, specifically through the action of genes encoded in the 52-kb polyketide synthase (pks) pathogenicity island [124]. Two strains of *Lactobacillus reuteri*, ATCC PTA 6475 and ATCC 53608, were found to reduce infection by enteropathogenic *E. coli* (EPEC). Although the exact mechanism is still unclear, it could be related to competitive exclusion, i.e., competition between probiotic and pathogenic strains for binding sites on the epithelial surface. It is conceivable that, by binding to the mucus layer, *L. reuteri* could create a stronger physical barrier against EPEC infection [99].

CRC is frequently associated with impairments to the immune system. TNF-α, IL-6, IL-1, and chemokines induce tumor growth by promoting angiogenesis and suppressing immune-mediated tumor elimination, while dendritic cells (DCs) and natural killer (NK) cells play a critical role in the early defense against cancer [125]. Probiotics can enhance innate immune functions, including the phagocytic activity of neutrophils and the cytotoxic activity of NK cells; indeed, such abilities might lie at the root of their anti-infectious or anticancer effects [126]. For example, strains of lactic acid bacteria (LAB) were reported to regulate the maturation of myeloid DCs, polarizing the subsequent T-cell activity toward Th1, Th2, or even T-reg responses. Additionally, in a colitis-associated model of CRC, oral administration of *Lactobacillus casei* BL23 protected against tumor development through the modulation of IL-22, a cytokine that promotes the proliferation of cancer cells, and the upregulation of caspase-7, a gene involved in apoptosis [101].

Pro-inflammatory cytokines provide critical protection against colorectal tumorigenesis, and probiotics have been found to mediate this role. For example, the anti-tumorigenic cytokine IL-18 promotes protective host immunity through the actions of CD8^+^ cytotoxic T cells (Tc), NK cells, and Th1-driven macrophage activation [127]. This cytokine is crucial for the homeostasis, mucosal repair, and proliferation/differentiation of intestinal epithelial cells, as well as the induction of goblet cell mucus production, the expression of tight junction proteins, and the secretion of anti-bacterial peptides that are essential in preventing CRC development. A recent study of aged IL-18-deficient mice found that *Lactococcus lactis* subsp. *cremoris* C60 restored T cell populations in small intestinal lamina propria, which led to a rebound in IFN-γ production in the CD4^+^ T cell population [102]. Similarly, the probiotic strains *Lactobacillus plantarum* and *Lactobacillus. salivarius* were able to augment IL-18 production in both in vitro and rat models of CRC [104]. In addition to LAB species, yeast is also able to immunomodulate IL-18 levels. In a CRC model, *Saccharomyces cerevisiae* was reported to have a pro-apoptotic effect via upregulation of the expression of IL-18; downregulation of the expression of TNF-α, IL-17, and IL-1β; and inactivation of the NF-κB and mTOR signaling pathways via downregulation of the target molecule, which is overactivated in CRC [128]. Probiotic strains are also able to modulate host immune functions through the production of derived molecules or cell envelope components [129]. For example, the administration of high-dose lysates of *L. acidophilus* significantly reduces the number of visible tumors and average body weight in colitis-associated CRC models. *L. acidophilus* lysates act as immunological adjuvants to activate the immune response; in particular, a significant enhancement was observed in subsets detected for Th1 helper lymphocytes (CD3^+^, CD4^+^, and IFN-γ^+^) and M1 macrophages (CD11b^+^, F4/80^+^, and CD86^+^) in mesenteric lymph nodes [91,92]. In a mouse model of AOM-induced CRC, oral consumption of the probiotics *L. acidophilus* and *Bifidobacterum bifidum* increased IFN-γ and IL-10 serum levels and the number of CD4^+^ and CD8^+^ cells. Administration of the probiotics inhibited the incidence of colonic lesions by about 57% for *L. acidophilus* and 27% for *B. bifidum* compared to the AOM-only group [95].

### Regulation in Apoptotic Genes in CRC by Probiotics

Another way in which probiotics can affect CRC is through the regulation of genes implicated in cell proliferation and apoptosis. One rarely studied example involves the modulation of microRNAs (miRNA), which can act as either oncogenes or tumor suppressors based on the cellular microenvironment where they are expressed. In a model of CRC induced by AOM/dextran sodium sulfate (DSS) treatment, levels of miR-155 (which induces resistance to chemotherapeutic agents) are dramatically increased [130]. The oral administration of *B. longum* to CRC mice resulted in a significant decrease in the elevated expression of miR-155, as well as that of the onco-miR miR-21a; moreover, in both healthy and CRC mice, treatment with *B. longum* increased levels of tumor-suppressing miR-145 and miR-15a. This probiotic treatment resulted in the downregulation of both NF-κB and miR-146a (which regulates IL-1β and IL-6 expressions) [105]. The probiotic’s effect on miR-21 is of particular interest given that the expression of miR-21 leads to enhanced cell proliferation, intravasation, cell migration, and metastasis, as well as declined rates of apoptosis, which, together, contribute to an enhanced cancer incidence and the diminished efficacy of drug therapies [131]. Furthermore, miR-21 is frequently upregulated in several kinds of carcinomas, for instance, colon and gastric cancers [132]. This suggests that drugs or supplements that can inhibit miR-21 might help in colon cancer treatment, as well as support the function and efficacy of chemotherapies.

A probiotic strain need not be alive to exert beneficial effects; dead probiotics or even cell components have been reported to effectively combat cancer. In a 2015 study by Lee et al. [103], a dead strain of *L. plantarum* inhibited AOM/DSS-induced colitis-associated carcinogenesis in mice better than the live bacterium did. This was reportedly due to the effects of inflammation suppression, apoptosis, and enhanced IgA secretion. AOM/DSS control animals possessed colon tumors, but administration with dead *L. plantarum* significantly suppressed the development of neoplasia by increasing the levels of secretory IgA. Specifically, it appeared that dead probiotics were more easily taken up by M cells than pure live probiotics were, thus generating a stronger secretory immune response.

A recent study on the probiotic *L. reuteri* demonstrated the anti-metastatic and antiproliferative effects of high-molecular-weight secretory molecules-cell-free supernatant components from heat-killed sonicated probiotic bacteria [96]. An exopolysaccharide (EPS) of *L. acidophilus* 20079 was noted to have a direct cytotoxic effect on tumor cells via mechanisms of apoptosis, stimulation of the immune response, and inactivation of the NF-κB inflammatory pathway. Extracted EPS from this strain may thus represent a promising therapeutic strategy for cancer [96]. Furthermore, EPSs produced by multiple strains of probiotic lactic acid bacteria, including *Lactobacillus plantarum* GD2, *Lactobacillus rhamnosus* E9, *Lactobacillus brevis* LB63 isolated from healthy infant feces, and *Lactobacillus delbrueckii* subsp. *bulgaricus* B3 isolated from yogurt, were reported to have an anticancer effect on colon cancer cells (HT-29). In this case, *Lactobacillus* EPSs were found to induce apoptosis in CRC in vitro through the increased expression of Caspase 3, Caspase 9, and BAX and decreased levels of Bcl-2, which led to a decline in cancer cell survival [100].

Some probiotics have been implicated in the inhibition of the epidermal growth factor receptor (EGFR) pathway, which can play an important role in CRC-related signaling. Some studies suggest that, during CRC, overexpression of the genes EGFR and HER-2 results in the deregulation of this pathway, leading to increased cell proliferation, prolonged cell survival, anti-apoptotic effects, and metastasis [133]. For this reason, these two genes are now potential targets for anticancer therapies such as cetuximab and trastuzumab (anti-CRC drugs) and anti-EGFR and HER-2 monoclonal antibodies, which are already available on the market. The related Notch and Wnt/β-catenin pathways have also been shown to be modulated by probiotics, in this case, by a cocktail of lactobacilli (L. cocktail), to generate antitumor effects in HT-29 cells in vitro. Specifically, the L. cocktail resulted in the Notch- or Wnt-induced promotion of apoptosis and the downregulation of cell proliferation. Therefore, the use of probiotic lactobacilli as nutritional supplements may both prevent colon cancer and represent a cost-effective and safe means of CRC treatment [107]. Generally, the pathogenesis of CRC is highly correlated with the deregulation of the Wnt/β-catenin signaling pathway. The major effector of the canonical Wnt signaling pathway is β-catenin (encoded by the CTNNB1 gene), which has a variety of cellular functions. In more than half of all cancer cases—including colorectal carcinoma, breast cancer, and liver carcinoma—nuclear localization of β-catenin always induces tumorigenesis and promotes the proliferation and survival of cancer cells [134].

In summary, some of the suggested mechanisms by which probiotics promote CRC prevention include the improvement of the host immune response, induction of apoptosis, and inhibition of tyrosine kinase signaling pathways [135,136].

## 7. Synbiotics

There has been a great deal of interest in the use of probiotics in combination with prebiotics, nonviable food components that confer health benefits on the host associated with modulation of the microbiota [137]. Gibson and Roberfroid [138] introduced the term “synbiotic” to describe the combination of synergistically acting probiotics and prebiotics. With respect to colorectal carcinogenesis, synbiotics have been demonstrated to have protective effects via multiple different mechanisms, including the modulation of the intestinal microbiota and immune response, reduction of inflammation, biosynthesis of compounds with antitumor activity, and improvement in the antioxidant system [139]. For example, the effects of co-administration of the probiotic VSL#3 and the prebiotic yacon (*Smallanthus sonchifolius*, a tuberous root rich in phenolic compounds and with a high soluble fiber content) were tested in a model of colitis-associated carcinogenesis. The synbiotic demonstrated numerous potential benefits: it supported the integrity of the intestinal barrier, increased the concentrations of SCFAs, as well as enzymes involved in the endogenous antioxidant defense system, and led to alterations in the general composition of the intestinal microbiota [111]. Furthermore, in a model of carcinogenesis induced by 1,2-dimethylhydrazine (DMH), treatment with VSL#3 and yacon reduced the size and number of pre-neoplastic lesions. In particular, the synbiotic was found to increase the secretion of IL-2 and IL-4; the former has effects on the regulation of immune cells and has been inversely correlated with tumor size, while the latter occurs concomitantly with the expression of TLR4, resulting in the improvement of the innate immune response and antitumor defense [111]. In a similar study, Lin et al. [97] demonstrated the protective effect of a combination of germinated brown rice (GBR) and *L. acidophilus*/*Bifidobacterium animalis* subsp. *lactis* in a DMH/DSS rat model. The synbiotic inhibited preneoplastic lesions (aberrant crypt foci) and decreased the activity of antioxidant enzymes (SOD) and apoptosis-related proteins in the colon (caspase-3 and Bcl-2). The authors hypothesized that, as GBR is a good substrate for certain colonic bacteria—and, thus, promotes fermentation and the production of SCFAs in the colon—the colonic epithelium may use the increased supply of SCFAs to produce additional mucin. In this way, the synbiotic may modulate the colonic secretion of mucins and their alterations during colorectal carcinogenesis to prevent the formation of more advanced aberrant crypt foci. Similarly, the consumption of yogurt containing *B. longum* (BB536-y) and fructo-oligosaccharides was found to enhance the amounts of SCFAs in fecal samples from healthy individuals. This combination significantly suppressed the amount of *Bacteroides fragilis* enterotoxin detected, and the SCFAs exerted a growth-inhibitory activity in human colon cancer cell lines [106]. Further evidence of the supportive effects of SCFA production have been found in studies of multiple probiotic strains. For example, treatment with the butyrate-producing bacterium *Clostridium butyricum* ATCC 19398 significantly inhibited intestinal tumor development in Apcmin/+ (Min, multiple intestinal neoplasia) mice by decreasing proliferation and increasing apoptosis. This strain suppressed the Wnt/β-catenin signaling pathway and modulated the gut microbiota composition, as demonstrated by decreases in some pathogenic and bile acid-biotransforming bacteria, and increases in some beneficial bacteria, including those that produce SCFAs [113]. An inhibited proliferation of colon cancer cells was also noted in response to the production of SCFAs, mostly propionic and butyric acid, by *Pediococcus pentosaceus* FP3, *L. salivarius* FP35, *L. salivarius* FP25, and *Enterococcus faecium* FP51 [114].

Another mechanism by which synbiotics have been documented to restore intestinal homeostasis in CRC is by improving antioxidant properties. For example, the administration of ginger extract, together with *L. acidophilus* strain MTCC 5401, had a positive effect on reducing gut inflammation (i.e., decreases in TNF-α and IL-6 levels) and decreasing the expression of the inflammation-associated genes Cox-2, iNOS, and c-Myc. Interestingly, treatment with either ginger extract or LAB alone failed to produce any effect on the antioxidant properties; together, however, they caused significant declines in the levels of malonaldehyde (MDA, a mutagen and tumor promoter) and significant increases in the levels of superoxide (SOD) and catalase (CAT), two important enzymatic antioxidants [93]. The antioxidant epigallocatechin gallate (EGCG) has demonstrated the potential for use as a prebiotic with *Lactobacillus* species because, unlike many bacteria, they possess the phenol decarboxylase and inducible acid phenol reductase activities that are necessary to metabolize phenolic acids such as EGCG [140]. Finally, a recent study of the combination of *Cudrania tricuspidata* leaf extract with *Lactobacillus gasseri* 505 reported that this synbiotic releases bioactive peptides from β-casein and phenolic compounds with antioxidant activities. In an AOM/DSS model, this treatment ameliorated the effects of cancer by downregulating pro-inflammatory cytokines (TNF-α, IFN-γ, IL-1β, and IL-6) and anti-apoptotic factors (Bcl-2 and Bcl-xL), and upregulating anti-inflammatory cytokines (IL-4 and IL-10) and pro-apoptotic factors (p53, p21, and Bax). Furthermore, synbiotic treatment decreased the expression of the inflammation-associated enzymes iNOS and COX-2 [115]. These effects were corroborated by work in a mouse model that demonstrated the ability of this combination to prevent the hepatic toxicity induced by CRC [116].

A particularly interesting example may be the combination of *Lactobacillus* and cranberries. Studies have indicated that phenolic compounds from cranberries act as antimicrobial substances against food pathogens such as *E. coli*; however, they do not exert an inhibitory effect on some *Lactobacillus* strains and may even act as growth-promoting factors for probiotics. Indeed, a combination of concentrated cranberry juice and cell walls extracted from a probiotic biomass (*L. acidophilus* CL1285, *L. casei* LBC80R, and *L. rhamnosus* CLR2) was found to exert an increased inhibitory effect against HT-29 cells. Furthermore, the phenolic compounds and probiotic biomass stimulated the activity of quinone reductase, a phase II detoxifying enzyme that offers protection against toxic and reactive chemical species [98].

## 8. Postbiotics

Postbiotics are the complex mixtures of metabolic products secreted by probiotics in cell-free supernatants—including enzymes, secreted proteins, short-chain fatty acids, vitamins, secreted biosurfactants, amino acids, peptides, and organic acids—that exert beneficial effects on the host, directly or indirectly [141]. As postbiotics do not contain live microorganisms, the risks associated with their intake are minimized. Postbiotics are conceptually similar to paraprobiotics, which are the inactivated microbial cells of probiotics (intact or ruptured, containing cell components such as peptidoglycans, teichoic acids, and surface proteins) or crude cell extracts (i.e., with complex chemical composition) [142]. A recent example focused on *L. rhamnosus* (LR) KCTC 12202BP, which is known to inhibit the cytokine-mediated apoptosis of mouse and human intestinal epithelial cells by regulating signaling pathways. In lysates, An et al. [108] identified an LR-derived therapeutic protein, p8, that suppressed CRC proliferation. This protein translocated specifically to the cytosol of DLD-1 (human CRC cell line) cells, where it downregulated the expression of Cyclin B1 and Cdk1 (p53-p21-Cdk1/Cyclin B1 signaling pathway), both of which are required for cell cycle progression. Another tumor-suppressive molecule, ferrichrome, was identified in conditioned media of the probiotic strain *L. casei* ATCC334 [143]. Ferrichrome is known to be a siderophore and a mediator of the bacterial anti-tumor function on colorectal cancer, inducing apoptosis by the activation of c-jun N-terminal kinase. Subsequently, the anti-tumor effect of ferrichrome was tested in an AOM-DSS model of carcinogenesis, where it was found to induce apoptosis via the upregulation of DDIT3 (DNA damage inducible transcript 3). However, ferrichrome did not demonstrate any anti-inflammatory activity in a DSS-mouse model, indicating that it inhibits cancer cell growth but not the advent of a precancerous condition such as inflammation [110].

The term ‘metabiotics’ refers to the functional metabolites secreted by probiotics that can optimize host-specific physiological functions; these are emerging as potential anticancer agents due to their ability to alter metabolic processes in the gut lumen and reduce the severity of colon carcinogenesis [144]. For example, a metabiotic extract from *L. rhamnosus* MD 14 demonstrated anticancer potential in the DMH rat model by reducing fecal procarcinogenic enzymes, oxidants, and aberrant crypt foci; downregulating numerous oncogenes (K-ras, β-catenin, Cox-2, and NF-κB); and upregulating tumor-suppressing p53. The metabiotic signature of *L. rhamnosus* MD 14 was characterized by several short-chain fatty acids (i.e., acetate, butyrate, and propionate), as well as other active compounds (i.e., acetamide, thiocyanic acid, and oxalic acid; [109]). *Lactococcus lactis* subsp. *lactis* produces a lantibiotic bacteriocin, nisin A, that was recently found to prevent the growth of cancer cells. Nisin demonstrated anti-metastatic effects on multiple colon cancer cell lines, including LS180, SW48, HT-29, and Caco-2. It was hypothesized that nisin’s probiotic effects might be caused by changes in intracellular calcium concentrations, which play an important role in apoptosis, specifically through downregulating the gene expression of carcinoembryonic antigen (CEA), carcinoembryonic cell adhesion molecule 6 (CEAM6), and two matrix metalloproteinases (MMP2 and MMP9) [112].

## 9. Next-Generation Probiotics

Recent studies have highlighted many potential next-generation probiotics (NGPs). These include *Prevotella copri* and *Christensenella minuta*, which control insulin resistance; *Parabacteroides goldsteinii, Akkermansia muciniphila*, and *Bacteroides thetaiotaomicron*, which reverse obesity and insulin resistance; *F. prausnitzii*, which protects mice against intestinal diseases; and *Bacteroides fragilis*, which reduces inflammation and exhibits anticancer effects [145]. In particular, *A. muciniphila* may have further potential for use in anticancer immunotherapy, such as treatments that target programmed cell death protein 1 (PD-1). Intriguing results were seen in an investigation of the differences in the microbiota of patients who responded to anti-PD1 therapy and those who did not. *A. muciniphila* was found to be particularly enriched in the microbiota of responders, and its importance was confirmed using fecal microbiota transplantation into germ-free mice. *A. muciniphila* was able, by itself, to improve the compromised efficacy of the anti-PD-1 blockade in mice that were given the microbiota from nonresponders. This finding has particular relevance given the increased popularity of cancer immunotherapy treatments aimed at the PD-1 protein and its ligand, PD-L1 (programmed death ligand 1), which have shown benefits in patients with various types of cancer [117]. Another next-generation probiotic currently under study is *Butyricicoccus pullicaecorum*, which can prevent necrotic enteritis and reduce pathogen abundance in the cecum and ileum, and has been reported to be safe in a human intervention trial [118]. This bacterium has also been linked to CRC, as it was found to be significantly less abundant in the stools of patients with late-stage CRC. The anticancer effects of *B. pullicaecorum* appear to be linked to its high production of butyrate, which was reported to inhibit CRC cell growth via the upregulation of SLC5A8 and GPR43 in an animal model of DMH/DSS tumorigenesis [119]. SLC5A8 and GPR43 are known to serve as tumor suppressors; mice lacking SLC5A8 develop CRC, while the activation of GPR43 prevents colon inflammation and carcinogenesis [146].

## 10. Discussion

CRC is one of the most common cancers in the world, affecting approximately 1 million people. The occurrence of CRC can have genetic or environmental origins but can also be due to a previously established disease (such as IBD). Another emerging factor that plays a part in CRC susceptibility is the composition of the intestinal microbiota. Indeed, the gut microbiota can affect many physiological functions involved in the control of epithelial cell proliferation and differentiation, prevention of pathogen growth, and stimulation of intestinal immunity. Many key discoveries regarding the role of the microbiota in CRC have originated from the use of axenic mouse models. For example, when conventional IL-10-deficient mice are administered AOM, they develop colitis and carcinomas in the colon, while axenic AOM-IL-10^−/−^ mice are tumor-free and without histological damage. Similarly, axenic AOM-IL-10^−/−^ mice colonized with *Bacteroides vulgatus* present more tumors than conventional mice [147]. In general, the microbiota of CRC patients has been shown to be different from that of healthy individuals, with the genera *Fusobacteria, Bacteroides*, and *Prevotella* being more predominant [41,148]. In addition, the mucosa of these patients presents higher levels of adherent-invasive *E. coli* [149]. Some strains are also known to have pro-carcinogenic characteristics and can initiate CRC onset [60]. For example, *Enterococcus faecalis* produces superoxide ions (O_2−_) that can be converted to hydrogen peroxide (H_2_O_2_), which causes DNA damage [150]. Some strains of *E. coli* produce the genotoxin colibactin, which induces cuts in DNA [56,61]; similarly, the production of a toxin by *B. fragilis* is responsible for the degradation of a tumor suppressor protein, E-cadherin, leading to cell proliferation and permeability of the intestinal barrier [151]. Altogether, these results demonstrate the strength of the association between alterations in gut microbiota composition and CRC.

Recent advances in our understanding of the composition of the microbiota have highlighted the implications of beneficial bacterial “probiotics” for human health. Currently, most of the anticancer effects of probiotic bacteria have been studied in vitro or using in vivo animal models. Together, these studies have revealed that the anti-CRC effects of probiotics arise through various mechanisms, including: (i) alteration of the composition of the microbiota, (ii) inactivation of carcinogenic compounds, (iii) competition with pathogenic or CRC-promoting bacteria, (iv) stimulation of the immune response, (v) regulation of apoptosis and cell differentiation, (vi) fermentation of undigested nutrients, and vii) pH acidification [136]. In humans, one of the best studied groups of probiotics is the lactic acid bacteria, which may prevent the development of CRC. Indeed, the consumption of dairy products containing *Lactobacillus* seems to be related to a low incidence of CRC [92,96,103,105,106,152]. In addition, epidemiological studies have shown that, even among individuals with a high-fat diet (which favors CRC development), the incidence of this cancer was lower in consumers of milk, yogurt, and other dairy products [153,154,155]. Furthermore, there is strong evidence that many of the anticancer effects of probiotic bacteria are mediated through their production of metabolites such as SCFAs [119]. Indeed, SCFAs (in particular, butyrate) can induce changes in apoptosis, cell cycle arrest, and cell differentiation; for this reason, there is currently a great deal of interest in the use of butyrate-producing bacteria, such as *F. prausnitzii*, to treat CRC [145].

To conclude, the microbial ecosystem of the intestine exerts a considerable influence on the human physiology through its metabolic and immune functions. A disturbance in intestinal homeostasis and in the gut microbiota can favor the appearance of certain pathologies, such as CRC. Due to the deep and fundamental links between these pathologies and the gut microbiota, the modulation of the species composition of these communities represents an attractive therapeutic alternative. In this context, the beneficial effects of probiotic bacteria on CRC have been well established, but studies have thus far been limited to only a few bacterial effectors and host molecular mechanisms. In parallel, a large number of studies have demonstrated the potential anticancer effects of probiotics in vitro or in vivo, but the evaluation of their curative effects on CRC has been more complicated. Recent studies in the technological advancement to analyze the human intestinal microbiota have established a new paradigm for the development of tools in the early detection of CRC through biomarkers. The identification of the exacerbation of a specific group of pathogenic bacteria or their metabolites will define a personalized strategy to counteract intestinal microbial dysbiosis. One of the strategies could be the use of probiotic strains to counteract intestinal microbial dysbiosis, one of the etiological agents of CRC and other intestinal disorders. To develop effective probiotic-based therapies against CRC, it will be essential to improve the characterization of the crosstalk between the microbiota and the host and to further elucidate the beneficial mechanisms of probiotics.

## Figures and Tables

**Table 1:** List of probiotics used in in vitro and in vivo CRC studies.

Microorganism	Function	Mechanism	Year	Reference
*acidophilus*	Reduction in tumor in colitis-associated CRC models	Activation of immune response by enhancing Th1 helper lymphocytes and M1 macrophages	2013	[91]
			2019	[92]
*L. acidophilus* strain MTCC 5401	Alleviation of gut inflammation	Decreasing the expression of the inflammation-associated genes; reducing the levels of TNF-α, IL-6, and malonaldehyde; increasing the levels of superoxide and catalase	2018	[93]
*L. acidophilus*	Protection against H. pylori	Inhibition of *H. pylori* adherence through the production of acetic acid and other bactericidal substances.	2019	[94]
*L. bulgaricus*		Prevention of TLR4/NF-κB signaling, and production of the IL-8 pro-inflammatory cytokine		
*L. acidophilus*	Inhibition of the incidence of colonic lesions	Elevation of IFN-γ and IL-10 serum levels and the number of CD4^+^ and CD8^+^ cells	2019	[95]
*B. bifidum*				
*L. acidophilus*	Cytotoxic effect on tumor cells	Stimulation of immune response, effect on apoptosis, and inactivation of NF-κB inflammatory pathway	2018	[96]
*L. acidophilus*	Prevention of the formation of advanced aberrant crypt foci and CRC	Inhibition of pre-neoplastic lesions and reduction in the activity of antioxidant enzymes (SOD) and apoptosis-related proteins (caspase-3 and Bcl-2)	2019	[97]
*B. animalis subsp. lactis*				
*L. acidophilus* CL1285	Protection against toxic and reactive chemical species and inhibition of colon cancer (HT-29) cell proliferation	Stimulation of quinone reductase activity	2020	[98]
*L. casei* LBC80R				
*L. rhamnosus* CLR2				
*L. reuteri*	Reduction of enteropathogenic *E. coli* (EPEC) infection	Creation of a strong physical barrier against EPEC infection by binding to the mucus layer	2016	[99]
*Lactobacillus* EPSs	Anticancer effect on colon cancer cells	Induction of apoptosis by increasing the expression of Caspase 3, Caspase 9, and BAX, and reducing the levels of Bcl-2	2019	[100]
*L. casei*	Protection against CRC development	Regulation of cancer cells proliferation and apoptosis through modulation of IL-22 and upregulation of caspase-7, respectively	2017	[101]
*L. lactis*	Prevention of CRC development	Restoration of T cell populations and regulation of IFN-γ production in the CD4^+^ T cell population	2020	[102]
*L. plantarum*	Inhibition of colitis-associated carcinogenesis	Suppression of inflammation and apoptosis, and elevation of IgA secretion	2015	[103]
*L. plantarum*	Prevention of CRC development	Upregulation of IL-18 production	2020	[104]
*L. salivarius*				
*B. longum*	Colon cancer treatment	Reduction in the elevated expression of miR-155 and onco-miR miR-21a, elevation in the levels of tumor-suppressing miR-145 and miR-15a, and downregulation in NF-κb and miR-146a	2019	[105]
*B. longum* (BB536-y)	Inhibition of CRC growth	Enhancement of SCFAs production and reducing the amount of *Bacteroides fragilis* enterotoxin	2018	[106]
*Lactobacilli cocktail*	Prevention and treatment of colon cancer	Modulation of Notch- or Wnt/β-catenin signaling pathway, apoptosis, and downregulation of cell proliferation	2020	[107]
*L. rhamnosus* KCTC 12202BP	Inhibition of intestinal epithelial apoptosis and suppression of CRC cell proliferation	Regulation of p53-p21-Cdk1/Cyclin B1 signaling pathway by downregulating the expression of Cyclin B1 and Cdk1	2019	[108]
*L. rhamnosus* MD 14	Anticancer effect	Reducing fecal procarcinogenic enzymes, oxidants, and aberrant crypt foci, downregulating numerous oncogenes, and upregulating tumor-suppressing p53	2020	[109]
*L. casei* ATCC334	Inhibition of CRC cell growth	Induction of apoptosis by upregulation of DDIT3	2021	[110]
VSL#3	Reduction in the size and number of pre-neoplastic lesions in a model of colitis-associated cancer	Regulation of the intestinal barrier integrity and endogenous antioxidant defense system by increasing the level of SCFAs and enzymes, and alterations in the general composition of the intestinal microbiota	2020	[111]
*L. lactis subsp. lactis*	Anti-metastatic effects on multiple colon cancer cell lines	Regulation of apoptosis by changing the intracellular calcium concentrations, and downregulating the expression of CEA, CEAM6, and matrix metalloproteinases (MMP2 and MMP9)	2018	[112]
*butyricum*	Inhibition of intestinal tumor development	Decreasing proliferation, increasing apoptosis, suppressing the Wnt/β-catenin signaling pathway, and modulating the composition of gut microbiota	2020	[113]
*P. pentosaceus* FP3	Inhibition of colon cancer cell proliferation	Production of SCFAs (propionic and butyric acid)	2013	[114]
*L. salivarius* FP35 and FP25				
*E. faecium* FP51				
*L. gasseri* 505	Improvement of CRC	Downregulating pro-inflammatory cytokines and anti-apoptotic factors, and upregulating anti-inflammatory cytokines and pro-apoptotic factors	2020	[115]
	Prevention of hepatic toxicity induced by CRC		2020	[116]
*A. muciniphila*	Cancer immunotherapy treatments	Improvement of anti-PD-1 blockade efficacy	2018	[117]
*B. pullicaecorum*	Prevention of necrotic enteritis and CRC	Reducing pathogen abundance in the cecum and ileum	2018	[118]
	Anticancer effect and inhibition of CRC cell growth	Production of butyrate and upregulation of SLC5A8 and GPR43	2020	[119]

## Data Availability

Not applicable.
